# Identification of Dynamic Changes in Proteins Associated with the Cellular Cytoskeleton after Exposure to Okadaic Acid

**DOI:** 10.3390/md11061763

**Published:** 2013-05-24

**Authors:** Jill A. Opsahl, Sonja Ljostveit, Therese Solstad, Kristin Risa, Peter Roepstorff, Kari E. Fladmark

**Affiliations:** 1 Proteomic Unit (PROBE), Department of Biomedicine, University of Bergen, Jonas Lies vei 91 N-5009, Norway; E-Mail: jill.opsahl@biomed.uib.no; 2 Department of Molecular Biology, University of Bergen, Thormolensgt 55 N-5008, Norway; E-Mails: sonja.ljostveit@mbi.uib.no (S.L.); kristin.risa@helse-bergen.no (K.R.); 3 The Biotechnology Centre, University of Oslo, Gaustad alleèn 21 N-0349, Norway; E-Mail: therese.saunders@legemiddelverket.no; 4 Department of Biochemistry and Molecular Biology, University of Southern Denmark, Odense 5230, Denmark; E-Mail: roe@bmb.sdu.dk

**Keywords:** okadaic acid, apoptosis, cytoskeleton, cell adhesion, phosphorylation, lipid rafts, quantitative proteomics

## Abstract

Exposure of cells to the diarrhetic shellfish poison, okadaic acid, leads to a dramatic reorganization of cytoskeletal architecture and loss of cell-cell contact. When cells are exposed to high concentrations of okadaic acid (100–500 nM), the morphological rearrangement is followed by apoptotic cell death. Okadaic acid inhibits the broad acting Ser/Thr protein phosphatases 1 and 2A, which results in hyperphosphorylation of a large number of proteins. Some of these hyperphosphorylated proteins are most likely key players in the reorganization of the cell morphology induced by okadaic acid. We wanted to identify these phosphoproteins and searched for them in the cellular lipid rafts, which have been found to contain proteins that regulate cytoskeletal dynamics and cell adhesion. By using stable isotope labeling by amino acids in cell culture cells treated with okadaic acid (400 nM) could be combined with control cells before the isolation of lipid rafts. Protein phosphorylation events and translocations induced by okadaic acid were identified by mass spectrometry. Okadaic acid was shown to regulate the phosphorylation status and location of proteins associated with the actin cytoskeleton, microtubules and cell adhesion structures. A large number of these okadaic acid-regulated proteins have previously also been shown to be similarly regulated prior to cell proliferation and migration. Our results suggest that okadaic acid activates general cell signaling pathways that induce breakdown of the cortical actin cytoskeleton and cell detachment.

## 1. Introduction

A number of algal toxins target the broad acting serine/threonine protein phosphatases (PP) 1 and 2A [1,2]. The toxins are produced both by cyanobacteria (microcystins and nodularin) and marine dinoflagellates (okadaic acid and dinophysistoxins) [3]. Intriguingly, these phosphatase inhibitors may act both as tumor promoters [4,5] and cell death inducers [6]. This dualistic counteracting effect seems to be dosage-dependent, both *in vivo* and *in vitro* [7]. Even though, human exposure to PP inhibiting algal toxins have a toxic or carcinogenetic effect on certain tissues and organs, due to primary exposure site or specific uptake mechanisms [8], as long as the toxins are able to enter vertebrate cells, their intracellular effect seems to be cell type-independent [6,9,10,11].

Cellular exposure to phosphatase inhibiting algal toxins leads to a rearrangement of the cytoskeleton and disruption of cell-cell interactions. The toxin-induced cytoskeletal rearrangement seems to be reversible at low dosage exposure [12,13], thus increasing cell motility and invasiveness, whilst high dosage exposure leads to cell death [14,15].

Exposure to okadaic acid and other PP-inhibiting toxins induces a reorganization of actin filaments, followed by changes in intermediate filaments and microtubules [11,16,17]. Okadaic acid also alters the properties and structures of proteins involved in cell-cell adhesion [18,19,20]. This reorganization requires a highly coordinated action of regulating proteins in which the detailed mechanisms are still unknown.

PP1 and PP2A control more than 90% of all serine/threonine dephosphorylation in mammalian cells, thus inhibition of these phosphatases results in phosphorylation of a large number of proteins [10,21]. We believe that proteins regulating the cytoskeletal reorganization and disruption of cell-cell interaction can be found among these phosphorylated proteins. The challenge is to enrich these low abundant phosphoproteins in order to be able to identify them using mass spectrometry.

The specialized membrane areas, called lipid rafts, have a central role in regulating cell-to-cell interaction through coupling the cytoskeleton to the cell membrane [22]. Even though cytoskeletal proteins do not directly interact with lipid membranes, they interact with proteins that associate with rafts of the inner leaflet of the plasma membrane [23,24]. Isolating the lipid rafts from toxin-exposed cells, therefore, appeared as an efficient way to enrich for proteins with key functions in regulating the observed cytoskeletal reorganization and disruption of cell-cell interaction that occur in cells exposed to PP-inhibitory toxins. Another important way to increase the chances of identifying regulating proteins was to have a synchronized and rapid cellular response. 

In this study, we use the diarrheic shellfish poison and PP-inhibitor, okadaic acid. Although okadaic acid is not classified as a neurotoxin, neurotoxic effects were early reported [25,26]. In the range of 100–500 nM, okadaic acid induces a rapid protein synthesis independent of cell-cell detachment, followed by cell death in neuroblastoma cell line, SH-SY5Y cells [27,28]. We therefore chose to expose the SH-SY5Y cells to a rather high concentration (400 nM) of the PP-inhibiting toxin, okadaic acid. By using stable isotopic labeling of amino acids in cell culture (SILAC) combined with mass spectrometry, we could mix okadaic acid- and vehicle-exposed SH-SY5Y prior to the isolation lipid rafts and enrichment of possible cytoskeleton-regulating proteins [29].

Here, we have combined SILAC labeling of the SH-SY5Y cell line with isolation of lipid rafts in order to identify phosphorylation and translocations of cytoskeletal-associated proteins in okadaic acid exposed cells. These events may be necessary for the observed okadaic acid-induced cytoskeletal reorganization, which precedes cell-cell detachment and apoptosis, to take place.

## 2. Results and Discussion

### 2.1. Actin and Morphological Re-Organization in SH-SY5Y Cells Exposed to Okadaic Acid

**Figure 1:**
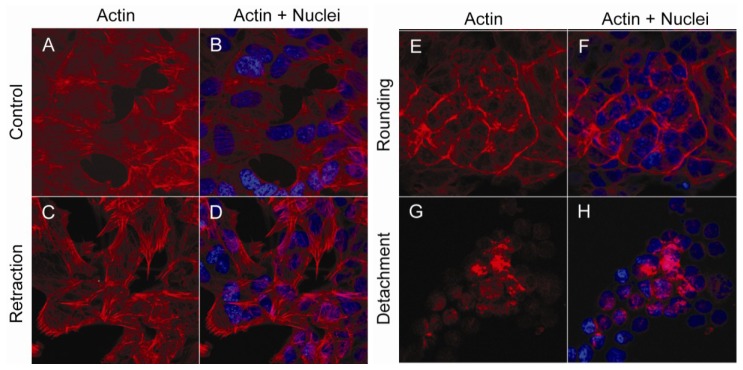
Actin re-organization in okadaic acid-exposed cells. SH-SY5Y cells were left untreated (**A**,**B**) or exposed to 400 nM okadaic acid for 25 (**C**,**D**) or 50 (**E**–**H**) min. Fixed cells were labeled with Rhodamine-conjugated phalloidin, and actin distribution (red) was visualized using confocal microscopy. Nuclei were labeled using DAPI (blue).

When SH-SY5Y cells were exposed to okadaic acid (400 nM), a rapid re-organization of filamentous actin could be observed (Figure 1). In the flattened-shaped monolayer of control cells, actin fibers stretched out through the cells and also covered the inner surface of the cellular membrane (Figure 1A,B). Twenty-five min after addition of okadaic acid, retraction fibers of actin were observed at the cell edges, as the cells started to round up (Figure 1C,D). Fifty minutes after addition of okadaic acid, cells had rounded up or detached from each other and the culture dish. At this stage, actin was either found concentrated at the inner surface of the cellular membrane (Figure 1E,F) or in polarized “clumps” in the floating cells (Figure 1G,H). At 75 min after addition of okadaic acid, the percentage of apoptotic cells, as judged by chromatin condensation, was 41 ± 4.

### 2.2. Enrichment and Identification of Okadaic Acid-Regulated Proteins Associated with the Cytoskeleton

**Figure 2:**
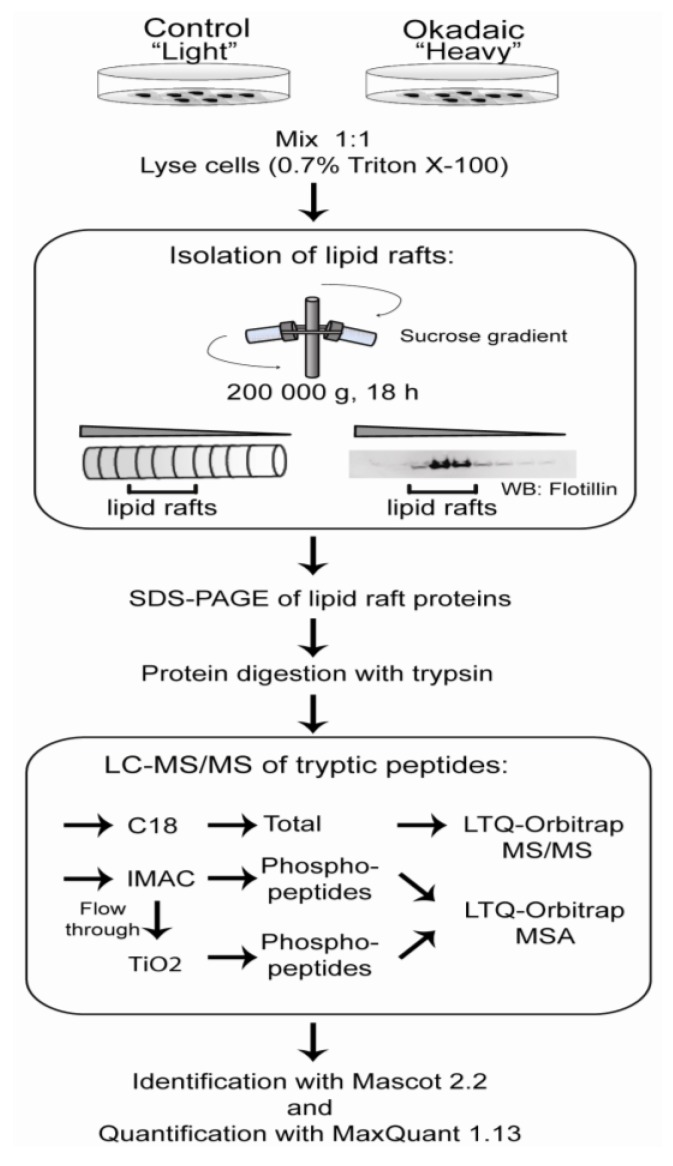
Workflow to identify lipid raft-associated proteins. Stable isotopic labeling of amino acids in cell culture (SILAC) labeled SH-SY5Y cells left untreated or exposed to 400 nM okadaic acid for 50 min before mixed in a ratio 1:1 in 0.7% Triton X-100/MES buffer. After centrifugation in a sucrose gradient, 10 fractions of 0.5 mL were collected. The lipid raft fractions were identified by Western blotting using flotillin as a lipid raft marker. Fractions containing flotillin were pooled prior to removal of lipids by chloroform/methanol extraction, followed by acetone precipitation. Proteins were separated by SDS-PAGE. Proteins were in-gel digested with trypsin, and peptides were purified either with (1) C18 or (2) IMAC/TiO_2_ and analyzed on LTQ-Orbitrap. Proteins were identified with Mascot (ver. 2.2) and quantified using MaxQuant (ver. 1.13).

To enable identification of proteins and phosphoproteins involved in okadaic acid-induced cytoskeletal reorganization and cell-cell disruption, we isolated lipid rafts, which are known to have important functions in cell adhesion and cytoskeletal organization [30]. Changes in the lipid raft proteome after okadaic acid exposure were determined using stable isotope labeling with amino acids in cell culture (SILAC) in combination with mass spectrometry. The workflow is depicted in Figure 2.

Our SILAC experiments with okadaic acid-exposed cells were divided into two parts: (i) Identification of lipid raft associated proteins with an altered phosphorylation status (Table 1); and (ii) Characterization of changes occurring in the lipid raft proteome in general (Table 2).

**Table 1:** Regulation of cytoskeleton-associated phosphoproteins in okadaic acid-exposed cells.

Accession ^a^	Protein	Entry name	Phosphopeptide sequence ^b^	P-site	Reference ^c^	Fold change ^d^
**Up-regulated**						
Q9NQP4	Prefoldin subunit 4	PFDN4	_FGS(ph)NINLEADES	S125	[31]	novel
A6NG51	Putative uncharacterized protein SPTAN1	SPTAN1	_S(ph)LQQLAEER	S1217	[32]	novel
P49006	MARCKS-related protein	MARCKSL1	_GEVPPKET(ph)PK	S38	[31]	1.9
P16949	Stathmin	STMN1	_ESVPEFPLS(ph)PPK	S38	[33]	1.6
Q15149	Plectin-1	PLEC1	_AQLEPVAS(ph)PAK	S1435	[32]	1.6
C9JDD6	Microtubule-associated protein tau	MAPT	_SPVVSGDT(ph)SPR	T739	[34]	1.6
P46821	Microtubule-associated protein 1B	MAP1B	_VQSLEGEKLS(ph)PK	S1779	[32]	1.4
			_ASVSPM(ox)DEPVPDSE S(ph)PIEK	S1389	[35]	1.4
			_SDIS(ph)PLTPR_	S1785	[32]	1.5
P23528	Cofilin-1	CFL1	_(ac)AS(ph)GVAVSDGVIK	S3	[32]	1.3
Q9C0C2	Tankyrase 1 binding protein 1, 182 kDa	TNKS1BP1	_VSGAGFS(ph)PSSK	S1138	[32]	1.3
			_NRS(ph)AEEGELAESK	S1666	[32]	1.3
**Down-regulated**						
Q01082	Spectrin beta chain	SPTBN1	_RPPSPEPS(ph)TK	S2106		52.1
O15021	Microtubule-associated Ser/Thr-protein kinase 4	MAST4	_DCPTLCKQ(de)TDNR Q (de)T(ph)DK	T2357		30.0
Q9Y4G6	Talin-2	TLN2	_LDEGT(ph)PPEPK	T1843	[32]	1.5
Q14155	Rho guanine nucleotide exchange factor 7	ARHGEF7	_M(ox)S(ph)GFIYQGK	S518	[36]	1.3

^a^ UniProt accession with a Mascot score >20 (*p* < 0.05); ^b^ Identified phosphopeptide with a PTM score >80; ^c^ Previously published identification of phosphorylation site; ^d^ SILAC-based fold change between okadaic acid-treated “heavy” cells and control “light” cells.

**Table 2:** Changes in lipid rafts associated cytoskeleton-regulating proteins after okadaic acid exposure.

Accession ^a^	Protein	Entry name	Fold change ^b^	# Peptides
**Up-regulated**				
P30153	Ser/Thr-protein phosphatase 2A regulatory subunit a	PPP2R1A	23	3
A8K0Y4	Putative uncharacterized protein GAP43	GAP43	2.2	9
Q12860	Contactin-1	CNTN1	1.9	5
O43707	Alpha-actinin-4	ACTN4	1.6	2
**Down-regulated**				
P07737	Profilin-1	PFN1	1.3	5
P35221	Catenin alpha-1	CTNNA1	1.3	4

^a^ UniPROT accession with a Mascot score >20 (*p* < 0.05); ^b^ SILAC-based fold change between okadaic; acid-treated “heavy” cells and control “light” cells.

### 2.3. Identification of Cytoskeleton-Associated Phosphoproteins Regulated by Okadaic Acid in SH-SY5Y Cells

**Figure 3:**
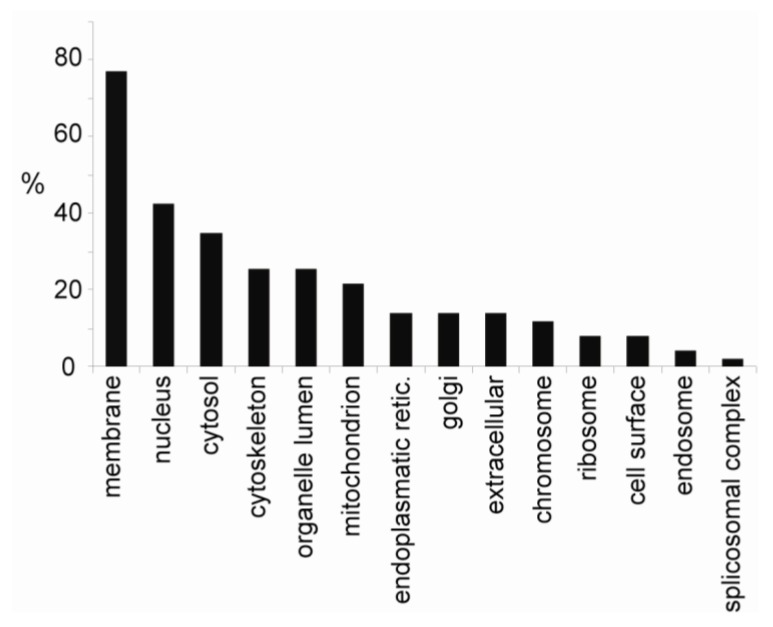
Distribution of the identified phosphoproteins according to cellular compartment. 167 unique phosphopeptides belonging to 67 proteins were categorized according to cellular compartment (GeneOntology). Note that a protein can be located in more than one cellular component.

From the SILAC-based experiments, we identified 167 unique phosphopeptides belonging to 67 proteins. A functional classification (Gene Ontology index-based), in which proteins may be found in more than one location, of the identified phosphoproteins is shown in Figure 3.

It should be noted that our use of 0.7% Triton X-100 results in less stringent conditions and a higher abundance of cytoplasmic proteins in the lipid raft preparation. Most importantly, we found that 23% of the identified phosphoproteins had previously been shown to be involved in regulation of the cytoskeleton and control of cell adhesion. Thirteen of these proteins were shown to be regulated, as defined by a 1.3-fold change or more [37] in cells after exposure to okadaic acid (Table 1).

The majority of these regulated phosphoproteins are known to be associated with the actin cytoskeleton: prefoldin subunit 4, MARCKS-related protein, plectin, cofilin-1, Rho guanine nucleotide exchange factor 7, talin-2, α-spectrin and β-spectrin [38,39,40,41,42]. Additionally, four microtubule-associated proteins were found to be regulated: stathmin, microtubule-associated protein 1B, microtubule-associated tau and microtubule-associated Ser/Thr kinase 4.

Some phosphopeptides/phosphoproteins were shown to be downregulated upon okadaic acid exposure. These were β-spectrin, microtubule-associated Ser/Thr-protein kinase 4, talin-2 and Rho guanine nucleotide exchange factor 7 (Table 1). Since okadaic acid inhibits Ser/Thr dephosphorylation, this downregulation most probably reflects a translocation or apoptosis-dependent cleavage of the phosphoproteins or that the specific identified phosphopeptides are further post-translationally modified and not picked up in our mass spectrometry analysis.

It should be noted that most of our identified phosphorylation sites have previously been observed in global phosphoproteome analysis [33,34]. Both these studies were performed in growth-stimulated cell lines. However, two phosphorylation sites: Ser2106 of β-spectrin and Thr2357 of microtubule-associated Ser/Thr-protein kinase 4, have, to our knowledge, not previously been reported. The ratio of phosphopeptides encasing these sites were found to decrease after okadaic acid exposure (Table 1).

Apart from being known to associate to either actin or microtubules, four of the okadaic acid-regulated phosphoproteins (talin-2, plectin-1, α-spectrin and MARCKS-related protein) are known to participate in the control of cell adhesion by linking the cytoskeleton to cell adhesion proteins [42,43,44].

### 2.4. Translocation of Cytoskeleton-Associated Proteins after Exposure to Okadaic Acid

SILAC-labeled cells were also used to characterize the effect of okadaic acid on the general lipid raft proteome. Our initial aim was to determine whether protein phosphorylation of cytoskeleton-associated proteins could result in translocation to or from the lipid rafts. However, only a small number of the proteins we found to be phosphorylated (Table 1) were also identified in the total lipid raft analysis. This reflects the complexity of the lipid rafts and also the need for phosphopeptide enrichment to enable analysis of cytoskeleton-associated phosphoproteins. On the other hand, we did observe okadaic acid-induced dynamic changes of proteins known to regulate the actin cytoskeleton and cell-cell adhesion (Table 2).

The most prominent upregulated protein in the lipid rafts after okadaic exposure was the regulatory subunit of PP2A (Table 2). Recently, it was shown that PP2A was also recruited to the lipid rafts in growth factor-stimulated cells [45]. The regulatory subunits of PP2A are believed to direct the holoenzyme PP2A to specific cellular localizations [46]. PP2A is a key player in the dynamic reorganization of cellular morphology. Even though its enzymatic function is inhibited by okadaic acid binding to its catalytic subunit, the cellular signal inducing translocation might still relocate PP2A.

**Figure 4:**
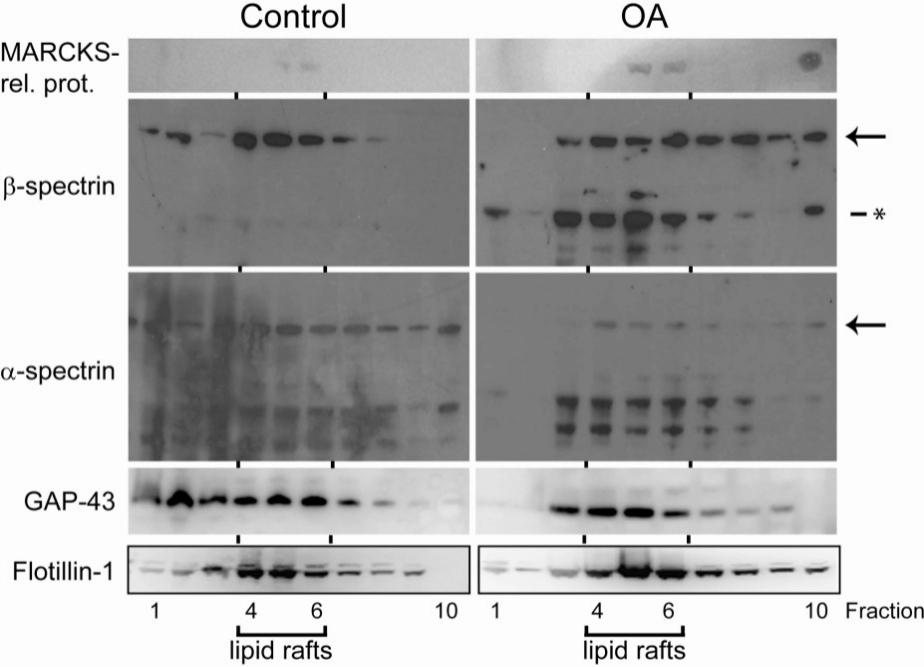
Verification of mass spectrometry-based results by Western blots of representative proteins. SH-SY5Y cells were treated with 400 nM okadaic acid for 50 min or left untreated two days after they had reached 100% confluency. Lipid rafts were isolated as described in the Materials and Methods sections, and proteins from all fractions (fractions 1–10) were separated by SDS-PAGE and immunoblotted using specific MARCKS-related protein, α-spectrin, β-spectrin, GAP43 and flotillin-1 antibodies. Arrows point to full-length spectrins and asterisks to the okadaic acid-induced cleavage product. Flotillin was used as a marker for lipid rafts (fractions 4–6).

The second most upregulated lipid raft-associated protein after okadaic acid exposure was GAP-43, an actin-binding protein known to be involved in the regulation of the cortical actin cytoskeleton [47]. This upregulation was also confirmed by Western blots, where we could observe that GAP-43 was recruited to the lipid rafts fractions from other cellular compartments in okadaic acid-exposed cells (Figure 4).

Furthermore, the amount of contactin-1 and α-actinin-4 was also found to be upregulated in response to okadaic acid treatment. Both proteins have a role in regulation of the actin cytoskeleton and cell adhesion and have also been shown to be associated with cell metastasis and cancer invasion [48,49,50].

**Figure 5:**
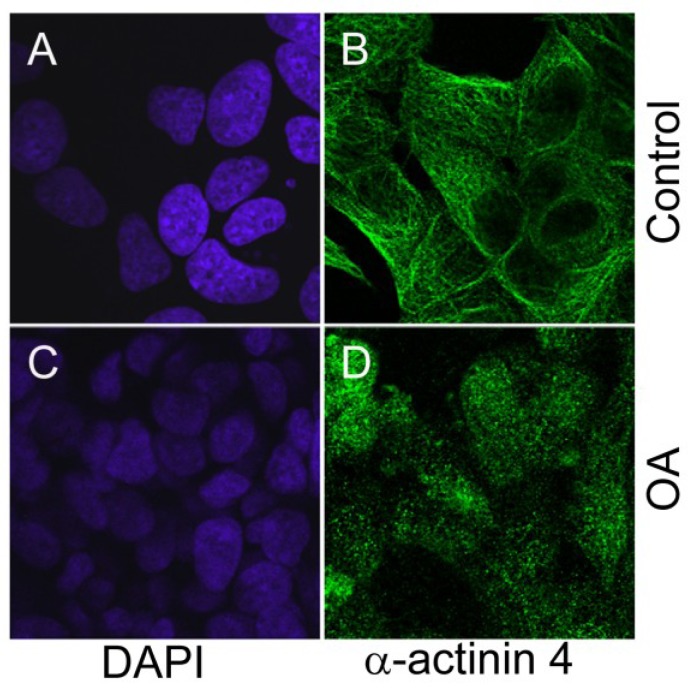
Okadaic acid disrupts the microfilament-associated structure of α-actinin-4 and induces cytoplasm to nucleus shuttling. Confocal images of SH-SY5Y cells left untreated (**A**,**B**); or exposed to 400 nM okadaic acid for 50 min (**C**,**D**). Cells were immunostained with anti-α-actinin-4 (**B**,**D**), and nuclei were labeled with DAPI (**A**,**C**).

A nuclear accumulation of α-actinin-4 has been observed in epidermal growth factor (EGF)-treated cells coinciding with its phosphorylation-dependent release from the actin filaments [51,52]. Based on these previous findings, we studied the cellular organization of α-actinin-4 before and after okadaic acid exposure using confocal microscopy (Figure 5). In flattened control cells, α-actinin-4 was found in “spots” alongside actin-like fibers (Figure 1) with increased amounts at the cell edges (Figure 5A,B). The nuclei were almost devoid of α-actinin-4. Okadaic acid disrupted this “fiber” organization of α-actinin-4 and increased the amount of nuclear α-actinin-4 (Figure 5C,D).

The actin-binding protein catenin-α-1 was found to be downregulated in the lipid rafts after okadaic acid exposure (Table 2). Catenin-α-1 is a linkage protein between cell-adhesion structures and the cortical actin cytoskeleton. Similar to actinin-4, its phosphorylation of catenin-α-1 promotes cancer cell invasion [53]. 

Also, the amount of the actin-binding proteins associated with lipid rafts decreased after okadaic acid treatment (Table 2). Profilin usually promotes actin polymerization [54]. Okadaic acid also increased Ser3 phosphorylation of cofilin-1 (Table 1), which has been reported to decrease its association to actin, which, in turn, enables actin depolymerization [38]. Taken together, the effect of okadaic acid on cofilin-1 and profilin would promote actin depolymerization.

### 2.5. Localization of Okadaic Acid-Regulated Phosphoproteins

Since only a limited number of peptides belonging to the okadaic acid-regulated phosphoproteins were identified in the general lipid raft proteome, Western blotting of lipid raft fractions were used to elucidate the localization of some selected okadaic acid-regulated phosphoproteins: MARCKS-related protein and α- and β-spectrin (Figure 4) (Table 1). 

The total amount of MARCKS-related protein, which showed an okadaic acid-dependent increase in Ser38 phosphorylation (Table 1), was found to increase in the lipid raft fractions (Figure 4). MARCKS-related protein gave weak signals by Western blotting and could only be observed in the lipid raft fractions.

The lipid raft associated full-length α- and β-spectrins decreased in okadaic acid-exposed cells (Figure 4). For full-length α-spectrin, a general decrease in all fractions (also non-lipid raft fractions) was observed. Similar to previous studies using SH-SY5Y cell lines, we also observed several lower-molecular weight forms of α-spectrin in control cells. However, we were not able to detect any increase related to okadaic acid exposure. In contrast, we did observe lower molecular weight fragments of β-spectrin after okadaic acid exposure (Figure 4). These β-spectrin fragments were mostly associated to the lipid raft fractions. Additionally, okadaic acid seemed to redistribute β-spectrin from the lipid rafts (Figure 4).

Taken together, the Western blots of lipid raft fractions supported that okadaic acid-induced phosphorylation of cytoskeleton-associated proteins (Table 1) also had an effect on their association to lipid rafts.

### 2.6. Okadaic Acid Induces a Coordinated Alternation in Proteins Associated with Cell Cytoskeleton and Cell Adhesion

**Figure 6:**
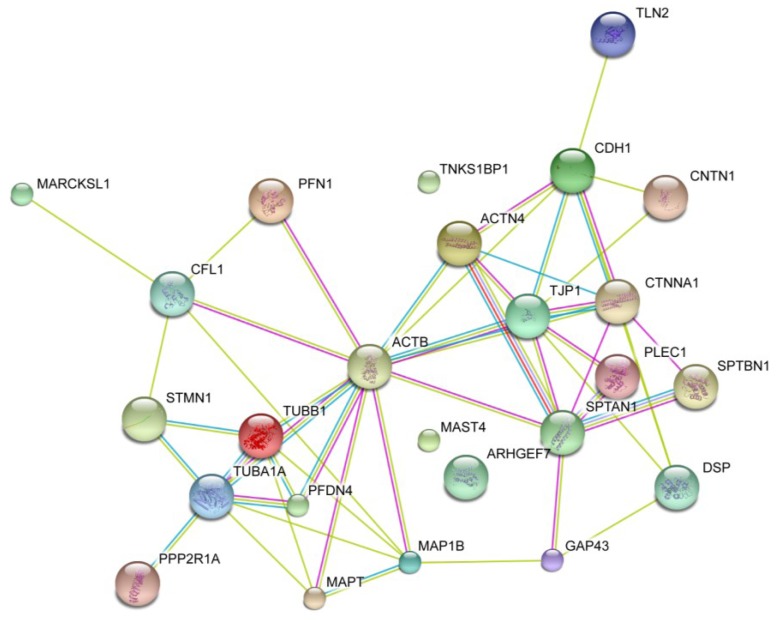
Protein interaction map of identified okadaic acid-regulated proteins. The map was constructed using the STRING 8.3 web tool [55]. Key proteins of cell adhesion platforms, cadherin (CDH1), tight junction protein (TJP1), desmoplakin (DSP), actin (ACTB) and tubulins (TUBA1A and TUBB1), were submitted to the database, together with the okadaic acid-regulated proteins (Table 1, Table 2). Colored lines denote interactions: green (text mining), red (gene fusion), blue (database) and grey (experiment).

Okadaic acid causes rounding of cells and detachment, which, in turn, depend upon cytoskeleton reorganization and disassembly of desmosomes, adherens junctions and tight junctions. To obtain a visual overview of physical and functional interactions between okadaic acid-regulated proteins (Table 1, Table 2), the cytoskeleton and proteins involved in cell attachment, we used String 8.3, a database of known and predicted protein interactions. Together with the lipid raft-associated okadaic acid-regulated proteins, we also submitted actin, tubulin and key proteins of attachments platforms (cadherin, desmoplakin and tight junction protein) to the interaction database (Figure 6). 

Apart from microtubule-associated Ser/Thr kinase family member 4 (MAST4), tankyrase-1 binding protein 1 (TNKS1BP1) and Rho guanine nucleotide exchange factor (ARHGEF7), all the identified okadaic acid-regulated proteins were shown to functionally interact with each other or the introduced cytoskeletal/adhesion proteins when analyzed through the STRING functional interaction database (Figure 6).

Okadaic acid-induced changes in cell morphology and cell-cell interaction require a coordinating action between proteins regulating cell adhesion and those regulating cytoskeleton dynamics. Not surprisingly, okadaic acid regulates both these groups of proteins (Table 1, Table 2 and Figure 6). We found that okadaic acid regulated the following cell adhesion associated proteins: α-catenin (CTNNA1), contactin (CNTN1), talin-2 (TLN2), plectin-1 (PLEC1), actinin-4 (ACTN4), α-spectrin (SPTAN1) and GAP43 (GAP43). All these proteins are known to directly or indirectly interact with cadherin (adherens junctions), plectin (desmosomes) or tight junction protein 1 (tight junctions) (Table 1, Table 2 and Figure 6). This shows that okadaic acid regulates proteins associated with all three cell-cell adhesion platforms. Some of these proteins, e.g., α-catenin-1, talin-2, α-spectrin and plectin, are also known to be linkage proteins between cell adhesion platforms and the actin cytoskeleton. We also found that okadaic acid increased phosphorylation of several other lipid raft-associated proteins known to regulate actin dynamics: cofilin-1 (CFL1), prefoldin subunit 4 (PFDN4), tau (MAPT), MARCKS-related protein (MARCKSL1) and microtubules-associated protein 1B (MAP1B). Again, some of this actin-regulating proteins, prefoldin subunit 4, tau and microtubules-associated protein 1B, are further associated to microtubules. Together with stathmin (STMN1), which also showed increased phosphorylation in response to okadaic acid, these proteins may play key roles in the observed okadaic acid-induced reorganization of the microtubules [17].

Even though okadaic acid-regulated proteins, microtubule-associated Ser/Thr-kinase 4, tankyrase 1 binding protein 1 and Rho guanine nucleotide exchange factor, were not verified as cytoskeletal regulatory proteins by the String functional interaction database, several lines of evidence do suggest that these proteins are also important factors in cytoskeletal dynamics and cell adhesion [39,56,57].

### 2.7. Okadaic Acid-Induced Phosphorylation and Relocalization of Cytoskeletal-Associated Proteins May Be General Events of Both Apoptosis-Induction and Cellular Proliferation

**Figure 7:**
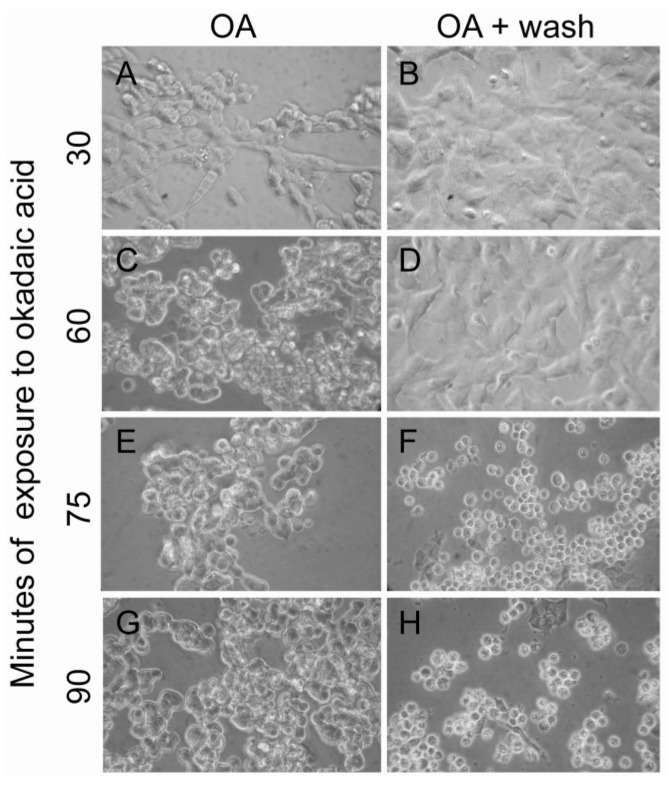
Okadaic acid-induced cell rounding is not directly coupled to cell death. Cells were exposed to 400 nM okadaic acid for 30, 60, 75 and 90 min before they were either fixed (**A**,**C**,**E**,**G**) or the toxin was removed (**B**,**D**,**F**,**H**). After removal of the toxin, cells were left to grow for two more days.

Okadaic acid can act as a double-edged sword, with the ability both to induce cell death and to increase proliferation and cell motility [7]. It is, therefore, interesting that the majority of the okadaic acid-regulated phosphorylation events associated to the cytoskeleton in apoptosis-induced cells (Table 1) also can be found in growth-stimulated cells (see references in Table 1). This supports the thought that these phosphorylation events are linked to cell rounding and cell-cell detachment and not necessarily to later occurring apoptosis [6,58]. If so, it should be possible to rescue okadaic acid-exposed cells from apoptotic cell death at a time point after cell rounding has occurred. We therefore removed okadaic acid from the cell culture after the cells had rounded up as a result to toxin exposure (Figure 7). After removal of the toxin, the cells were left to grow for two days. The rescue studies revealed that the commitment point to okadaic acid-induced cell death was at a time point after the cells had rounded. The okadaic acid-regulated protein phosphorylation and translocations of cytoskeleton associated proteins we observe may therefore be general events that are needed for cell rounding and detachment, e.g., also for proliferation and cell migration.

It should be noted that our main focus was to identify okadaic acid-induced alternations of the cytoskeleton-associated proteome that precede cell-cell detachment and apoptosis. These high-dosage exposure-related events seem protein synthesis-independent [6]. It is, therefore, important to consider that the low-dosage exposure and tumor promoting effects of okadaic acid may also involve altered gene transcription. Indeed, lower dosage experiments have also observed altered expression of genes and proteins associated with the cytoskeleton [59,60].

## 3. Experimental Section

### 3.1. Chemicals and Antibodies

Anti-α-actinin 4 (ab32816) was from Abcam (Cambridge, UK). Anti-MARCKSL1 (sc-130471), anti-GAP-43 (sc-7458), anti-α-spectrin (sc-53900) and anti-β-spectrin (sc-7467) were from Santa Cruz (Dallas, TX, USA). Anti-flotillin-1 (610821) was from BD Bioscience (San Jose, CA, USA). Okadaic acid was purchased from LC Laboratories (Woborn, MA, USA). All other biochemicals, if not indicated, were from Sigma-Aldrich (St. Louis, MO, USA).

### 3.2. Cell Culture

For SILAC-labeling human SH-SY5Y neuroblastoma cells were cultured in custom-made Dulbecco’s modified Eagle’s medium (DMEM) (Invitrogen 52100) depleted of the essential amino acids, arginine and lysine, supplemented with 10% dialyzed fetal bovine serum (Invitrogen 26400-044), penicillin (50 units/mL), streptomycin (50 mg/mL) and 2 mM glutamate. The “light” culture was supplemented with ^12^C_6_-Arg/Lys, and the “heavy” culture was supplemented with ^13^C_6_-Arg/Lys. After reaching 100% confluency, the cells were kept in culture for 48 h. The “heavy” culture was incubated with 400 nM okadaic acid for 50 min. Cells were harvested after washing with 1 × PBS (20 mM Na_2_HPO_4_, 2 mM NaH_2_PO_4_, 0.137 M NaCl, pH 7.4) and incubation for 5 min with PBS in which they easily detached. The cell pellet was stored at −80 °C.

For other experiments, SH-SY5Y cells were cultured in normal DMEM supplied with 1% penicillin (50 units/mL), streptomycin (50 mg/mL) and 10% fetal bovine serum. 

### 3.3. Actin Labeling

To visualize actin filaments, the cells were grown on coverslips and fixed in 2% formaldehyde two days after reaching 100% confluence. Blocking was done with 1% BSA in PBS and permeabilization with 0.1% Triton X-100 in PBS for 5 min. Cells were then washed 3 times with PBS, incubated with Rhodamine-conjugated phalloidin (final concentration 5 units/mL) in 1% BSA in PBS for 1.5 h in dark and washed 3 times with PBS, followed by labeling the nucleus with DAPI (1 μg/mL in PBS) for 5 min. Finally, cells were washed twice with PBS and mounted on glass slides.

### 3.4. Evaluation of Apoptosis

For assessment of apoptosis, cells were fixed in 4% formaldehyde with 1 μg/mL of the DNA-specific dye Hoechst 33342. Apoptosis was scored based on the appearance of chromatin condensation using a fluorescence microscope. Three hundred cells were evaluated per sample.

### 3.5. Lipid Raft Isolation

The protocol for lipid raft isolation was adapted from Parkin *et al.* [61]. All steps of the protocol were performed at 4 °C. Cells from “light”- and “heavy”-labeled cultures were combined and lysed in 800 μL 0.7% Triton X-100/MNE buffer (25 mM MES pH 6.5, 5 mM EDTA, 150 mM NaCl) by passing the cells suspension through a Luer 21G needle 15 times, followed by centrifugation at 16,000× *g* for 15 min. The supernatant was adjusted to 40% (w/v) sucrose by the addition of an equal volume of 80% (w/v) sucrose in MNE buffer. One milliliter cell lysate was layered beneath a discontinuous sucrose gradient consisting of 2 mL of 35% (w/v) sucrose and 2 mL of 5% (w/v) sucrose in MNE buffer. The sample was centrifuged at 200,000× *g* for 18 h. Sucrose gradients were harvested from the base of the tubes in 0.5 mL fractions. Western blot analysis of all fractions was performed on trichloroacetic acid precipitated aliquots. Based on flotilin-1 distribution, lipid raft containing fractions were added up to 5 mL with MNE and spun at 200,000× *g* for 2 h. Supernatant was discarded and the pellet was stored at −80 °C. 

### 3.6. Removal of Lipid and Protein Separation

The pellet was re-suspended in 100 μL 100 mM *N*-octyl-beta-d-glucopyranoside/100 mM Tris pH 7.8 and lipids removed by methanol/chloroform extraction. The resulting pellet was dissolved in 4X SDS sample buffer, alkylated for 20 min at room temperature with iodoacetamide (20 mM) and proteins separated by SDS-PAGE. For quantitative proteomics analysis, the gel was stained with Sypro Ruby (BioRad, Herkules, CA, USA) and scanned on a Typhoon (GE Healthcare, Princeton, NJ, USA).

### 3.7. Sample Preparation for Mass Spectrometry

The gel was sliced into equal sized bands. Proteins were in-gel digested with trypsin (Promega, Madison, WI, USA). Phosphopeptides were enriched by IMAC (Qiagen, Hilden, Germany) [62] and TiO_2_ (GL Science, Tokyo, Japan) [63] prior to LC-MS/MS analysis. For general lipid raft proteome analysis, peptides were desalted with homemade C18 StageTip (3M, St Paul, MN, USA).

### 3.8. Peptide Separation and Mass Spectrometry Analysis

For phosphopeptide analysis, peptides were separated on an Easy-nLC (Thermo, Bremen, Germany) coupled to an LTQ-Orbitrap XL (Thermo Scientific) using an in-house packed fused silica column (18 cm, 100 μm inner diameter, 375 μm outer diameter packed with ReproSilPur C18-AQ, 3 μm (Dr. Maisch, Ammerbuch, Germany). 

Settings for LC were: 0.25 μL/min flow rate; solvent gradient: 0% B–34% B in 30 min, then from 30% B to 100% B in 10 min. Solvent A was aqueous: 0.1% formic acid; solvent B was organic: 0.1% formic acid in 90% acetonitrile. Peptide masses were measured in the Orbitrap at a resolution of 60,000 (*m*/*z* 400). Up to five of the most intense peptides (intensity ≥15,000 counts) were selected from each MS scan and fragmented using multistage activation in the linear ion trap [64].

For general lipid raft proteome mapping, the peptides were separated on a Dionex Ultimate 3000 nano-LC system (Sunnyvale, CA, USA) coupled to a linear quadrupole ion trap (LTQ-Orbitrap) mass spectrometer (Thermo Scientific). Settings for liquid chromatography separation were: flow rate (nano column): 0.2 μL/min; solvent gradient: 5% B to 60% B in 42 min, then from 60% B to 95% B in 10 min. Solvent A was aqueous 2% acetonitrile in 0.1% formic acid, and solvent B was organic 80% acetonitrile in 0.1% formic acid. Peptides were separated on a 100 mm-long column packed with ReproSilPur C18-AQ (Dr. Maisch, Ammerbuch, Germany). 

### 3.9. Data Processing and Identification

The raw files were processed and quantified with Maxquant ver 1.0.13 using doublet labeling R/K C13 and a maximum of three labeled amino acids [65]. Mascot search files were generated from MaxQuant and searched by Mascot ver 2.2 with database comparison with human entries from the UniProt database [66]. The general search criteria were: carbamidomethyl as fixed modification, phosphorylation (STY), oxidation (M), deamidation (Q) as variable modification, two missed cleavage sites for trypsin, peptide delta mass tolerance ± 10 ppm and MS/MS tolerance ± 0.8 Da. All peptides were searched against IPI human (ver. 3.69) concatenated with the reverse database. Settings for quantification of SILAC pairs: Only razor or unique peptides unmodified, except for oxidation (M) and acetylation (protein *N*-term) protein, were used for protein quantification. The minimum ratio count was set to 1. The site false discovery rate (FDR) was 0.01, whereas the protein FDR and the peptide FDR were 0.05 and 0.02, respectively. In addition, only peptides with a Mascot score equal to *p* ≤ 0.05 were accepted. Peptides with individual fold change >1.3 where considered regulated [37].

ProteinCenter (Thermo) was used to categorize proteins according to GOindex. The String 8.3 database [66] was used to construct a visualization of the protein interaction network of our identified proteins.

### 3.10. Western Blotting

Proteins were separated on SDS-PAGE and transferred to a PVDF membrane using 14 V for 17 h at 4 °C. The membrane was blocked with 1% BSA for 1 h at room temperature. Primary antibody was incubated for 1 h at room temp in 1× PBS/Tween and incubated with secondary antibody (host-specific) after a 3 × 5 min wash with 1× PBS/Tween. Blots were developed using ECL from Pierce and imaged with Las 1000/3000 or Kodak film. 

### 3.11. Immunolabeling

Cells were fixed in 4% formaldehyde, permeabilized with 0.1% Triton X-100 and stained with anti-α-actinin 4, followed by FITC-conjugated antibodies (Jackson ImmunoResearch Laboratories, West Gove, PA, USA), respectively. Nuclei were stained with DAPI, and cells were viewed using a Zeiss LMS 510 Meta confocal scanning microscope.

### 3.12. Cell Death Rescue

Cells were incubated with 400 nM or vehicle for 30, 60, 75 or 90 min. At these time points, cells were either fixed in 4% formaldehyde or okadaic acid was removed. Toxin was removed by collecting the culture media and washing the cells twice with fresh media. Detached cells were collected, washed and added back to the cell culture. The cells were grown for 48 h after okadaic acid was removed.

## 4. Conclusion

We have identified cytoskeleton- and cell adhesion-associated proteins that are regulated prior to apoptotic cell death in okadaic acid-exposed neuroblastoma cells. The majority of these regulated proteins have previously also been shown to be regulated by phosphorylation in proliferating and migrating cells. Removal of okadaic acid at a time point after the cells had rounded up could rescue the cells from cell death. Thus, okadaic acid seems to activate, through inhibiting dephosphorylation, a general mechanism that initiates cell rounding and cell detachment. These are cellular events necessary for apoptotic cell death, but also for cell proliferation and motility. Our results may therefore give new insight on how okadaic acid may act as both a cell death inducer and also as a tumor promoter.
